# Epidemiological trends and patterns of Hodgkin lymphoma among Saudis

**DOI:** 10.3389/fonc.2026.1847489

**Published:** 2026-06-22

**Authors:** Abdulrahman Algarni, Abdullah Alshammari, Ezeldine K. Abdalhabib

**Affiliations:** 1 Department of Medical Laboratory Technology, Faculty of Applied Medical Sciences, Northern Border University, Arar, Saudi Arabia; 2 Department of Clinical Laboratory Sciences, College of Applied Medical Sciences, Jouf University, AlQurayyat, Saudi Arabia

**Keywords:** epidemiology, Hodgkin lymphoma, incidence, lymphoma, Saudi Arabia, Saudi cancer registry, trend

## Abstract

**Introduction:**

Hodgkin lymphoma is one of the most common malignancies reported in Saudi Arabia and is curable if detected early. An analysis of the epidemiology of Hodgkin lymphoma in Saudi Arabia may help identify epidemiological trends of the disease and, consequently, highlight potential areas for targeted interventions.

**Materials and methods:**

A retrospective population-based trend study was conducted to analyze data on the number of cases, age at diagnosis, gender, morphological subtypes, stage at diagnosis and regional distribution from 2011 to 2020, as published by the Saudi Cancer Registry (SCR). Descriptive statistical analyses were performed to evaluate temporal trends in incidence, stage distribution, morphological subtypes, and regional variation. Age-standardized incidence rates (ASR) were calculated using direct standardization. Temporal trends were assessed using log-linear regression to estimate the average annual percent change (APC).

**Results:**

A total of 4,563 cases of Hodgkin lymphoma were recorded over the span of the 10-year surveillance period. The proportion of Hodgkin lymphoma cases increased from 3.1% to 4.1% of cancer cases that were reported. The ASR increased from 1.8 to 2.7 per 100,000 individuals, accompanied by a statistically significant APC of +5.08% (p < 0.001). Male subjects exhibited a higher APC of +5.60% compared to females (+3.38%). A bimodal age distribution was observed, peaking at 15–19 years in males and 20–24 years in females. Morphologically, HL Not Otherwise Specified (NOS) showed a substantial increase from 13.6% to 56.2%, surpassing Nodular Sclerosis as the predominant subtype by the year 2020. While diagnoses at a distant stage remained prevalent, localized-stage diagnoses increased from 29.8% to 37.5% by 2018. Najran recorded the highest regional APC of +18.4%, whereas Madinah exhibited a notable and statistically significant decline of -4.3%.

**Conclusion:**

There is an increasing proportion of cancer cases attributable to Hodgkin lymphoma. The rising incidence of Hodgkin lymphoma is characterized by a shift toward the NOS (Not Otherwise Specified) subtype and significant geographical disparities. Furthermore, a trend toward diagnosis at earlier clinical stages suggests potential improvements in early detection.

## Introduction

Hodgkin lymphoma (HL) is a distinctive malignant neoplasm of B-cell origin. It is characterized by the presence of Hodgkin and Reed–Sternberg (HRS) cells, which are large, multinucleated cells within the tumor microenvironment (TME). This complex microenvironment consists of a small fraction of HRS cells and a diverse array of immune and stromal cells (1). Cancer currently accounts for approximately one in six deaths (16.8%) globally and nearly one in four deaths (22.8%) attributable to non-communicable diseases (NCDs) (2). Although HL accounts for only 0.4% of all cancers globally, it holds particular epidemiologic importance due to its bimodal age distribution, histological heterogeneity, and favorable prognosis with appropriate therapy (3).

The disease exhibits a bimodal age distribution, with incidence peaks in young adulthood (15–19 years) and late adulthood (≥55 years). Globally, HL demonstrates a slight male predominance and marked geographical variation, with higher incidence rates reported in the developed regions of Europe and North America compared to the less developed regions of Asia and Africa (4). In developed regions, nodular sclerosis classical HL (NSCHL) is more common, whereas mixed cellularity classical HL (MCCHL) predominates in developing countries (5). These variations are considered to be influenced by environmental, genetic, and viral factors, particularly the prevalence of Epstein–Barr virus (EBV) infection (6). Advances in chemotherapy and radiotherapy have resulted in improved treatment outcomes, with five-year relative survival rates reported as 85–90% in high-income countries and ranging from 66% in Thailand to 95% in Finland, thus highlighting the disparity in treatment outcomes between developing and developed countries (7–9).

In Saudi Arabia, HL was the fourth most common malignancy as reported in 2022 and had an estimated 5-year prevalence rate of 20.1 per 100,000 population (4). As such, the Saudi Cancer Registry (SCR) was established in 1992 under the Ministry of Health of the Kingdom of Saudi Arabia. This registry is a national, population-based cancer surveillance system that collates data from regional cancer registries located in the administrative regions of Saudi Arabia, which follow standardized case definitions and data collection protocols established by the International Agency for Research on Cancer (IARC) and the World Health Organization (WHO) to enable comparisons across regions. The registry is considered to be overall accurate, although there are a few gaps in data completeness. We proceeded with this study, considering that this was the only available reliable source of data (10–12).

According to the Saudi Cancer Registry Report (2020), 8.9% of all newly diagnosed cancers were lymphomas, with HL contributing to 4.1% of these cases (13). Between 2001 and 2020, an increasing trend in the incidence of HL was reported in males and in individuals under 40 years of age, with an estimated annual percentage change (APC) of 2.9% in males and 3.7% in females (14). There were sex-specific and region-specific variations, as well as variations in the morphological subtype and stage of diagnosis of HL across Saudi Arabia (15).

However, published national data disaggregated by morphology, stage, and age group remain limited. Despite advances in treatment, population-based survival data on HL in Saudi Arabia remain limited. Existing studies are largely institution-based and focus on generating clinical insights, but do not describe the demographic shifts, morphological transitions, and geographic heterogeneity (16–19). Such data are critical for evaluating disease burden and identifying populations at risk in order to guide national cancer control strategies. The present study aims to analyze the epidemiological patterns of HL in Saudi Arabia from 2011 to 2020.

## Methodology

### Study design

A retrospective population-based trend analysis study using secondary data.

### Source of data

Data were extracted from reports of the Saudi Cancer Registry (SCR) over the years 2011 to 2020 (20–29). The SCR is a population-based registry that compiles cancer data from all regions of Saudi Arabia using a standardized format. The SCR constitutes a national, population-based cancer registry that adheres to uniform protocols for cancer registration procedures and coding systems. The SCR was approached to obtain data on the number of cases, age and sex distribution, stage distribution and geographical location. The data were coded, and no personal identifiers were released with the data. All such data were compiled into a Microsoft Excel spreadsheet. This data was checked for duplications and missing data. Any missing data were assigned a value of ‘zero’ and were not considered for analysis. Access to the data was password-protected and restricted to the authors only. Since no personal identifiers were released with the data, clearance from the ethics committee was waived in accordance with institutional guidelines.

### Study setting and population

This analysis was conducted using secondary data from all HL cases reported among the Saudi national population residing in the administrative regions of Saudi Arabia between 2011 and 2020.

### Data extraction

A predefined Microsoft Excel spreadsheet was used to extract data from the available dataset. Data were extracted on the number of cancer cases, the number of HL cases, morphological subtypes of HL cases, stage distribution at diagnosis, incidence rates and age-standardized incidence rates (ASRs) by gender, age group and region within Saudi Arabia. Data were also extracted on the indicators of crude incidence rate (CIR) and age-specific incidence rate (AIR), disaggregated by gender and region. These data were extracted over a period of 10 years, from 2011 to 2020.

### Data analysis

Crude incidence rates (CIR) of HL were computed by dividing the number of new cases by the estimated mid-year Saudi population and presenting the results per 100,000 people. To account for age variations, age-specific incidence rates (AIRs) were additionally computed for five-year age groups. Age-standardized incidence rates (ASRs) were calculated using the direct standardization method with Segi’s World Standard Population as the reference and presented per 100,000 population (30). The 95% confidence intervals for the ASRs were calculated using a method which relies on assuming a Poisson distribution for counts in age groups (31). HL incidence trends were studied via log-linear regression, with annual ASR treated as the dependent variable and calendar year as the independent variable. Male-to-female rate ratios (RRs) were computed to measure the difference in incidence between the two sexes. The annual percentage change (APC) estimates were obtained through log-linear regression models. Regional changes were studied by fitting separate models for the 13 administrative regions, except for regions with fewer than three non-zero observations. Statistical analyses were carried out using Microsoft Excel, Python, and SPSS; the significance level was set at 0.05 for all statistical tests.

### Ethics statement

This research employed publicly accessible, anonymized, and aggregated data obtained from the Saudi Cancer Registry. In accordance with both institutional and national protocols, studies utilizing secondary, non-identifiable data are exempt from ethical review and informed consent requirements.

## Results

### Trends in the proportion of HL amongst cancer cases from 2011-2020

**Figure 1:**
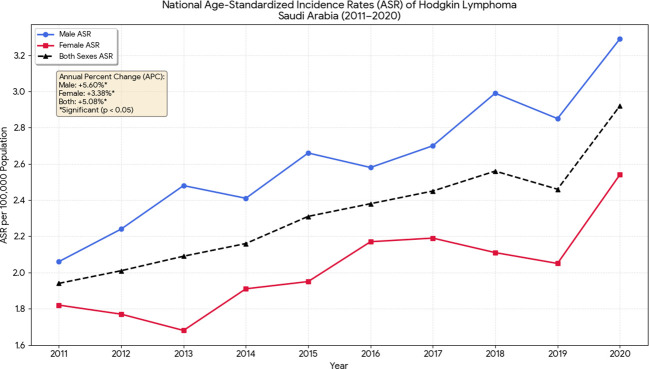
Trends in national age-standardized incidence rates (ASR) of HL per 100,000 population in Saudi Arabia, by Sex, 2011–2020.

During the ten-year surveillance period (2011–2020), a total of 4,563 cases of HL were recorded among 130,181 cancer cases, with year-to-year variations observed in both absolute counts and proportional incidence (**Table 1**; **Figure 1**). In 2011, 341 cases of HL were reported, representing 3.1% of all cancer cases (n = 10,869). The annual number of cases demonstrated a gradual upward trend, reaching a peak in 2020 with 581 cases, accounting for 4.1% of all reported cancer cases (n = 14,050). The largest relative increase was observed between 2015 and 2016, when the proportion of HL cases rose from 3% to 4.1%. The national ASR for both sexes exhibited an increase from 1.8 per 100,000 (95% CI: 1.79–2.09) in the year 2011 to 2.8 per 100,000 (95% CI: 2.25–3.66) by 2020. A statistically significant average APC of +5.08% (95% CI: +3.48% to +6.70%, p < 0.001) was documented for the aggregate population. Throughout the period analyzed, male incidence consistently surpassed female incidence. The male ASR increased from 1.9 to 3.3 per 100,000, resulting in a significant APC of +5.60% (p < 0.001). In contrast, the female ASR rose from 1.7 to 2.2 per 100,000, with a comparatively lower yet significant APC of +3.38% (p = 0.012). The male-to-female rate ratio (RR) ranged from 1.12 in 2011 to a peak of 1.67 in 2013. Male predominance was statistically significant in 8 out of the 10 years analyzed in the study.

**Table 1:** National age-standardized incidence rates (ASR) of Hodgkin lymphoma per 100,000 population, by sex and year, 2011–2020.

Year	Total cases	% of all cancers	Male ASR	Female ASR	Mean ASR	Rate ratio (95% CI)	p-value
2011	341	3.1	1.9	1.7	1.8	1.12 (0.91–1.39)	0.297
2012	355	3.2	2.0	1.6	1.8	1.25 (1.01–1.54)	0.036*
2013	393	3.4	2.5	1.5	2.0	1.67 (1.36–2.04)	< 0.001*
2014	411	3.5	2.3	1.8	2.1	1.28 (1.05–1.56)	0.013*
2015	436	3.6	2.6	1.7	2.2	1.53 (1.26–1.86)	< 0.001*
2016	495	3.8	2.6	2.3	2.5	1.13 (0.95–1.35)	0.175
2017	517	3.7	2.8	2.2	2.5	1.27 (1.07–1.51)	0.007*
2018	544	3.5	3.1	2.0	2.6	1.55 (1.30–1.84)	< 0.001*
2019	490	3.0	2.8	1.8	2.3	1.56 (1.30–1.87)	< 0.001*
2020	581	4.1	3.3	2.2	2.7	1.50 (1.27–1.77)	< 0.001*

* Indicates p < 0.05 (statistically significant).

### Age and sex distribution of HL cases

Age-specific incidence rates (AIR) exhibited a distinct bimodal distribution over the decade (**Table 2**). In the male population, HL cases showed a peak incidence in the 15-19-year age group, reaching a maximum AIR of 4.9 in 2018, followed by a second peak in the 50-54-year age group. In females, this peak occurred approximately five years later, in the 20–24-year age group, characterized by a peak AIR of 4.7 during the 2016–2017 period. A subsequent peak was observed in late adulthood. There was a notable sharp spike observed in the 0-4-year age group among males in 2020, reaching an AIR of 1.7 per 100,000, which represented the highest value recorded during the study period for this age group. In the male population, incidence rates increased significantly in the 65-69-year age group (up to 6.9 per 100,000) and in the ≥75-age group (peaking at 8.3 in 2017).

**Table 2:** Age-specific incidence rate (AIR), age-standardized incidence rate (ASR) among Saudi males and females (per 100,000) by age groups. .

Year	2011		2012		2013		2014		2015		2016		2017		2018		2019		2020	
Age Group	F	M	F	M	F	M	F	M	F	M	F	M	F	M	F	M	F	M	F	M
All Ages	157	184	161	194	154	239	176	235	169	267	230	265	229	288	207	337	183	307	234	347
0-4	0.3	0.3	0.3	0.2	0.2	0.2	0.2	0.4	0.1	0.3	0.4	0.4	0	0.7	0.1	0.3	0.3	0.2	1.1	1.7
5-9	0.4	0.4	0.5	0.6	0.5	1.1	0.9	2.1	0.7	1.6	0.8	2.2	0.7	1.2	0.4	1.3	0.7	1.5	1	2.5
10-14	1	1.5	1	1.3	0.7	2.1	2.2	2	1.9	2.2	1.4	2.5	1.9	2.7	1.4	3.2	1.6	2.7	2.4	3
15-19	2.4	2.8	3.4	3	2.8	3.9	3.9	3.6	2.6	4.7	4.6	2.7	3.9	4.8	4.1	4.9	2.9	4.8	3.2	3.1
20-24	3.8	3.6	2.7	3.1	3.2	3.2	2.5	3.9	3	4.1	4.7	3.2	4.7	2.7	4	4.4	3.1	2.8	3.3	2.9
25-29	2.5	2.5	2.8	2.5	1.8	4.3	2.8	3.1	2.6	2.9	3.4	3.4	2.7	3.3	3.2	4.8	2.8	3.4	2.7	4.1
30-34	0.9	2.3	1.3	2	2.3	2.4	2.4	2.4	1.8	3.3	2.6	4.4	3.7	3.1	2.8	3.6	2.1	4.3	2.8	3.2
35-39	1	1.8	1.7	2.3	1.2	1.6	0.7	2	1.2	2.4	1.3	2.1	1.6	3.1	1.4	3.6	1.3	3.1	2.1	3.1
40-44	1.9	1.9	0.4	1.9	0.5	1.8	0.7	1.6	1	2.2	1.9	0.9	2.1	2.4	0.6	3.6	0.5	3.1	2	1.3
45-49	0.7	1.4	0.9	2.7	1.8	1.7	1.6	1.1	1.2	2.2	1.8	2.8	2.5	2.9	1.1	2.1	0.7	2.8	1.5	3.3
50-54	1.2	2	1.8	4	0.9	2.7	1	3.1	2	3	2	3.9	1.2	2.5	0.5	2.9	2.1	3.8	2.7	5.6
55-59	2.5	1.2	2.4	2.3	3	3.6	2.3	2.1	2	1.2	3.6	3	0.3	3.2	3.4	4.9	1.2	2	1.5	5.6
60-64	3.4	2.2	2.2	2.6	0.5	2.5	1.8	1.3	4.4	3.7	2.2	2	5.2	2.8	3	2.8	5	1.2	2.5	3.1
65-69	1.5	0.8	2.2	2.2	3.5	2.8	2.6	0.7	0.6	2	1.3	2.7	1.9	4.6	3.1	2.6	3	5.1	3	6.9
70-74	7.7	4.6	4.2	3.3	4.1	9.7	1.8	4.7	3.7	6.5	4.5	6.5	0.9	3.6	2.6	6.3	0.9	1.8	0.8	4.3
75+	2.5	7.4	1.6	3.3	2.3	3.2	3.2	6.1	3.1	4	2.5	3.9	4.3	8.3	4.8	3.1	3.5	8	1.1	5.4
Crude Rate	1.6	1.9	1.6	1.9	1.5	2.3	1.8	2.3	1.7	2.6	2.3	2.6	2.3	2.8	2	3.2	1.8	2.9	2.2	3.2
ASR World	1.7	1.9	1.6	2	1.5	2.5	1.8	2.3	1.7	2.6	2.3	2.6	2.2	2.8	2	3.1	1.8	2.8	2.2	3.3

### Morphological distribution of Hodgkin disease in Saudi Arabia among males and females

The histopathological framework of HL underwent a significant transition between 2011 and 2020 (**Table 3**). Hodgkin lymphoma, Not Otherwise Specified (NOS), demonstrated an upward trend, reaching 56 cases in 2020, and emerged as the predominant subtype among males. The proportion of these cases decreased from 50% in 2011 to 23.3% in 2020 among males and from 51.6% to 32.5% among females. Hodgkin lymphoma, Nodular Sclerosis, remained the most common subtype in 2014. Hodgkin lymphoma, Mixed Cellularity, showed fluctuations over the study period but an overall decline, decreasing from 15.2% cases in 2011 to 8.6% cases in 2020 among males.

**Table 3:** Morphological distribution of Hodgkin disease in Saudi Arabia among males and females by subtype and year.

Morphological subtype (ICD-O-3)	Gender	2011 n (%)	2012 n (%)	2013 n (%)	2014 n (%)	2015 n (%)	2016 n (%)	2017 n (%)	2018 n (%)	2019 n (%)	2020 n (%)	Total
Nodular Sclerosis (9663)	Male	92 (50%)	90 (46.4%)	102(42.7%)	118(50.2%)	124(46.4%)	123(46.4%)	126(43.8%)	149(44.2%)	111(36.2%)	81 (23.3%)	1116
	Female	81 (51.6%)	101(62.7%)	88 (57.1%)	111(63.1%)	93 (55%)	129(56.1%)	133(58.1%)	103(49.8%)	84 (45.9%)	76 (32.5%)	999
NOS (9650)	Male	25 (13.6%)	30 (15.5%)	36 (15.1%)	35 (14.9%)	49 (18.4%)	42 (15.8%)	60 (20.8%)	94 (27.9%)	108(35.2%)	195(56.2%)	674
	Female	26 (16.6%)	19 (11.8%)	18 (11.7%)	25 (14.2%)	32 (18.9%)	48 (20.9%)	45 (19.7%)	57 (27.5%)	63 (34.4%)	120(51.3%)	453
Mixed Cellularity (9652)	Male	28 (15.2%)	39 (20.1%)	39 (16.3%)	36 (15.3%)	35 (13.1%)	38 (14.3%)	39 (13.5%)	48 (14.2%)	31 (10.1%)	30 (8.6%)	363
	Female	20 (12.7%)	20 (12.4%)	22 (14.3%)	10 (5.7%)	29(17.2%)	33(14.3%)	27 (11.8%)	22 (10.6%)	16 (8.7%)	19 (8.1%)	218
Lymphocyte-rich (9651)	Male	10 (5.4%)	8 (4.1%)	11 (4.6%)	7 (3.0%)	13 (4.9%)	14 (5.3%)	14 (4.9%)	9 (2.7%)	10 (3.3%)	9 (2.6%)	105
	Female	11 (7.0%)	1 (0.6%)	5 (2.6%)	6 (3.4%)	2 (1.2%)	9 (3.9%)	3 (1.3%)	4 (1.9%)	4 (2.2%)	4 (1.7%)	49
Nodular Lymphocyte Predominance (9659)	Male	16 (8.7%)	15 (7.7%)	23 (9.6%)	26 (11.1%)	10 (5.9%)	40 (15.1%)	41 (14.2%)	32 (9.5%)	41(13.4%)	31(8.9%)	275
	Female	5(3.2%)	4 (2.5%)	6 (3.9%)	18 (10.2%)	6 (3.6%)	5 (2.2%)	17 (7.4%)	16 (7.7%)	11 (6.0%)	12 (5.1%)	100
Lymphocyte depletion (9653)	Male	3 (1.6%)	0 (0.0%)	7 (2.9%)	6 (2.6%)	3 (1.1%)	2 (0.8%)	1 (0.3%)	4 (1.2%)	2(0.7%)	1 (0.3%)	29
	Female	4 (2.5%)	2 (1.2%)	3 (1.9%)	2 (1.1%)	1 (0.6%)	2(0.9%)	1 (0.4%)	2 (1.0%)	0 (0.0%)	0 (0.0%)	17
Nodular Sclerosis, grade 1(9665)	Male	4 (2.2%)	5 (2.6%)	16 (6.7%)	3 (1.3%)	4 (1.5%)	2(0.8%)	0 (0.0%)	0 (0.0%)	1(0.3%)	0 (0.0%)	35
	Female	3 (1.9%)	5 (3.1%)	7 (4.5%)	1 (0.6%)	0 (0.0%)	0 (0.0%)	2 (0.9%)	1(0.5%)	2 (1.1%)	1 (0.4%)	22
Nodular Sclerosis, grade 2 (9667)	Male	5(2.7%)	6 (3.1%)	4 (1.7%)	2 (0.9%)	3 (1.1%)	3 (1.1%)	3 (1.0%)	0 (0.0%)	2(0.7%)	0 (0.0%)	28
	Female	2 (1.3%)	5 (3.1%)	5 (3.2%)	2 (1.1%)	1 (0.6%)	4 (1.7%)	1 (0.4%)	1 (0.5%)	1 (0.5%)	0 (0.0%)	22
Nodular Sclerosis, cellular phase (9664)	Male	1 (0.5%)	1 (0.5%)	0 (0.0%)	0 (0.0%)	3 (1.1%)	1 (0.4%)	3 (1.0%)	1(0.3%)	0 (0.0%)	0 (0.0%)	10
	Female	3 (1.9%)	2 (1.2%)	1 (0.6%)	1 (0.6%)	1 (0.6%)	0 (0.0%)	0 (0.0%)	1 (0.5%)	2 (1.1%)	2(0.9%)	13
Lymphocyte depletion, diffuse fibrosis (9654)	Male	0 (0.0%)	0 (0.0%)	0 (0.0%)	0 (0.0%)	0 (0.0%)	0 (0.0%)	0 (0.0%)	0 (0.0%)	0 (0.0%)	0 (0.0%)	0
	Female	1 (0.6%)	0 (0.0%)	0 (0.0%)	0 (0.0%)	0 (0.0%)	0 (0.0%)	0 (0.0%)	0 (0.0%)	0 (0.0%)	0 (0.0%)	1
lymphocyte depletion, reticular (9655)	Male	0 (0.0%)	0 (0.0%)	0 (0.0%)	1(0.4%)	0 (0.0%)	0 (0.0%)	0 (0.0%)	0 (0.0%)	1 (0.3%)	0 (0.0%)	2
	Female	1 (0.6%)	0 (0.0%)	0 (0.0%)	0 (0.0%)	0 (0.0%)	0 (0.0%)	0 (0.0%)	0 (0.0%)	0 (0.0%)	0 (0.0%)	1
Hodgkin sarcoma (9662)	Male	0 (0.0%)	0 (0.0%)	0 (0.0%)	1 (0.4%)	00 (000%)	00 (000%)	1 (0.3%)	0 (0.0%)	0 (0.0%)	0 (0.0%)	2
	Female	0 (0.0%)	0 (0.0%)	0 (0.0%)	0 (0.0%)	0 (0.0%)	0 (0.0%)	0 (0.0%)	0 (0.0%)	0 (0.0%)	0 (0.0%)	0
Other/Unspecified	Male	0 (0.0%)	0 (0.0%)	0 (0.0%)	0 (0.0%)	0 (0.0%)	0 (0.0%)	0 (0.0%)	0 (0.0%)	0 (0.0%)	0 (0.0%)	0
	Female	0 (0.0%)	2 (1.2%)	0 (0.0%)	0 (0.0%)	0 (0.0%)	0 (0.0%)	0 (0.0%)	0 (0.0%)	0 (0.0%)	0 (0.0%)	2
Total		341	355	393	411	436	495	517	544	490	581	4563

Among females, HL NOS, and Hodgkin lymphoma, nodular lymphocyte predominance demonstrated an upward trend between 2011 and 2020, with incidence increasing by approximately from 16.6% to 51.3% cases and 59.4% (from 3.2% to 5.1% cases), respectively. In contrast, the number of Hodgkin lymphoma, lymphocyte-rich cases declined by 51.9% (from 5.4% to 2.6% cases) over the same period. A complete reduction (100%) was observed in Hodgkin lymphoma, lymphocyte depletion, which declined from 2.5% cases in 2011 to zero cases in 2020 (**Table 3**).

### Stage distribution of HL among Saudi nationals

**Figure 2:**
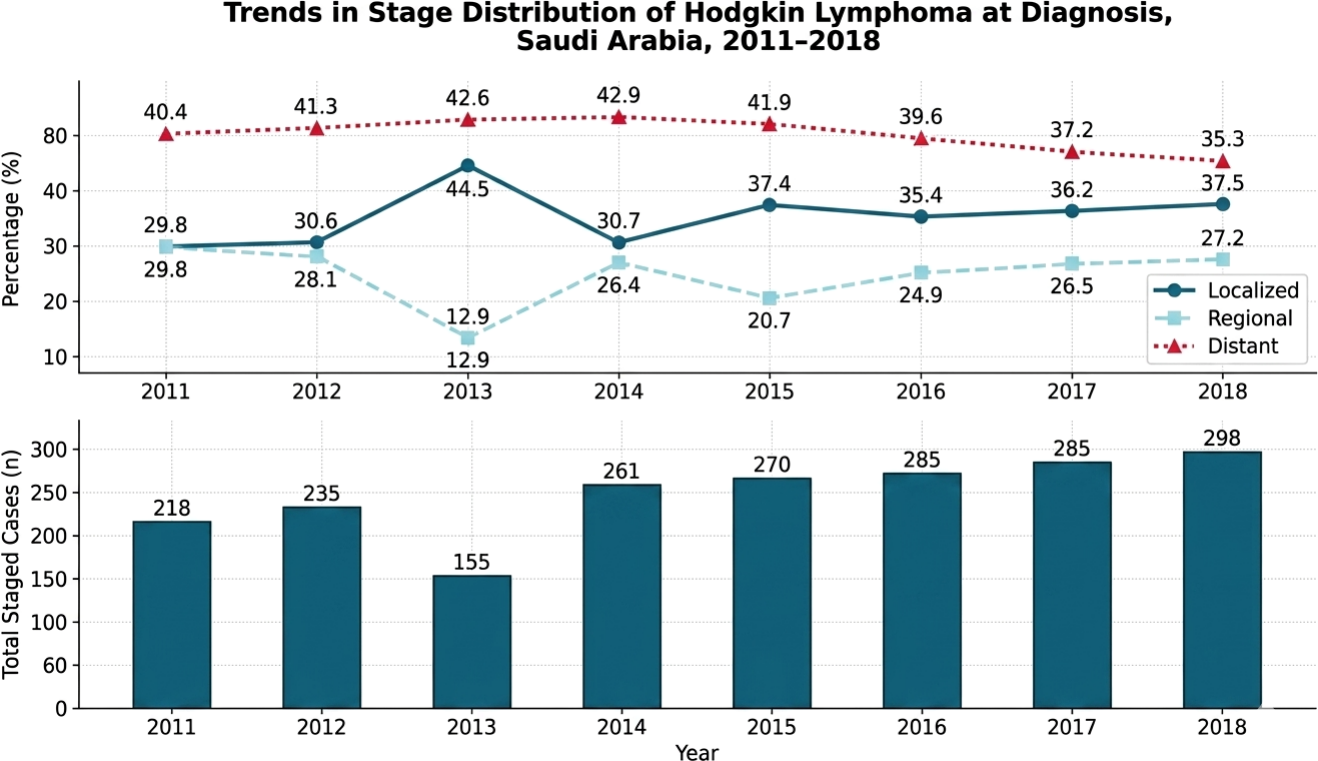
Trends in stage distribution of HL.

Across all stages, the distant stage of HL was most prevalent in 2014, with 43.1 reported cases, followed by the localized stage in 2015, with 37.3 cases. Data for the years 2019 and 2020 were unavailable. Throughout the study period, the regional stage consistently demonstrated the lowest prevalence, with the minimum observed in 2013 at 20 cases (**Figure 2**). There was a reduction in the proportion of cases diagnosed at the distant stage and an increase in those diagnosed at the localized stage, indicating earlier diagnosis.

### Regional distribution of HL in Saudi Arabia

Regional analysis highlighted substantial geographical heterogeneity (**Table 4**). Najran recorded the highest rate among males during the study period (7.4 per 100,000 in 2019) and had the highest APC (+18.4%; p = 0.009). The ASR of HL incidence among both sexes in Najran increased from 0.8 in 2011 to 5.0 in 2020. Statistically significant increases were also noted in Makkah (APC + 6.2%, p = 0.009), Riyadh (APC + 4.9%, p < 0.001), and the Eastern Province (APC + 4.9%, p = 0.006). Among females, Tabuk reported the highest incidence during the 2011–2013 period (ASR = 2.73), whereas Hail showed the lowest ASR (0.47) during the same period. In males, Jouf recorded the highest ASR during the 2017–2020 period (5.33). Madinah was the only administrative region to demonstrate a statistically significant decline in HL incidence, with an APC of −4.3% (95% CI: −7.9% to −0.7%, p = 0.026). Jazan consistently exhibited the lowest incidence rates, with the male ASR decreasing to 0.45 during the 2017–2020 period.

**Table 4:** Number of cases, age-standardized incidence rate (ASR) and crude incidence rate (CIR) of Hodgkin lymphoma by region, sex, Saudi Arabia, 2011–2020.

Region		2011–2013		2014–2016		2017–2020		Regional APC (95% CI) [p-value]
		Male	Female	Male	Female	Male	Female	
Asir	*Cases*	24	29	65	42	98	55	+7.0% (-2.4% to +17.5%) [p=0.129]
	*ASR*	1.00	1.37	2.23	1.93	2.73	1.55	
	*CIR*	0.97	1.13	2.57	1.57	2.77	1.52	
Baha	*Cases*	6	6	16	10	15	6	+1.0% (-10.1% to +13.4%) [p=0.850]
	*ASR*	1.17	1.23	1.53	2.63	1.85	0.70	
	*CIR*	1.10	1.03	2.40	1.73	1.95	0.75	
Tabuk	*Cases*	23	26	29	19	60	31	+0.5% (-8.0% to +9.8%) [p=0.902]
	*ASR*	2.37	2.73	2.83	1.73	3.88	2.08	
	*CIR*	2.13	2.53	2.57	1.87	3.90	2.12	
Jouf	*Cases*	9	11	15	8	37	21	+9.1% (-3.5% to +23.4%) [p=0.139]
	*ASR*	1.77	2.00	1.90	2.33	5.33	2.30	
	*CIR*	1.60	2.00	2.50	1.47	4.70	2.73	
Eastern	*Cases*	122	77	126	111	238	164	+4.9% (+1.9% to +8.1%) [p=0.006]
	*ASR*	3.00	1.70	2.73	2.33	3.68	2.68	
	*CIR*	2.60	1.70	2.63	2.50	3.55	2.65	
Madinah	*Cases*	40	33	45	32	43	35	-4.3% (-7.9% to -0.7%) [p=0.026]
	*ASR*	2.27	1.73	2.03	2.37	1.55	1.28	
	*CIR*	2.00	1.67	2.23	1.57	1.52	1.25	
Riyadh	*Cases*	179	123	202	158	362	231	+4.9% (+2.7% to +7.1%) [p=< 0.001]
	*ASR*	2.50	1.83	2.63	2.50	3.65	2.50	
	*CIR*	2.57	1.83	2.87	2.40	3.62	2.48	
Makkah	*Cases*	130	98	163	109	285	206	+6.2% (+1.9% to +10.6%) [p=0.009]
	*ASR*	2.10	1.53	2.23	1.93	3.00	1.75	
	*CIR*	2.00	1.50	2.43	1.67	3.03	2.25	
Qassim	*Cases*	33	31	44	37	71	45	+3.2% (-4.7% to +11.7%) [p=0.385]
	*ASR*	2.17	1.67	3.23	2.20	3.40	2.15	
	*CIR*	2.23	1.87	2.93	2.57	3.42	2.23	
Jazan	*Cases*	23	18	19	12	13	21	-8.0% (-16.6% to +1.4%) [p=0.083]
	*ASR*	1.33	1.17	1.03	0.63	0.45	0.83	
	*CIR*	1.33	1.00	1.03	0.70	0.53	0.85	
Najran	*Cases*	7	9	16	14	23	19	+18.4% (+5.7% to +32.6%) [p=0.009]
	*ASR*	0.63	1.03	3.20	1.77	3.00	2.45	
	*CIR*	0.50	0.93	2.50	2.20	2.52	2.12	
Hail	*Cases*	16	6	16	17	18	13	+5.5% (-5.3% to +17.5%) [p=0.285]
	*ASR*	1.77	0.47	2.20	1.40	1.58	1.00	
	*CIR*	1.97	0.50	2.07	2.13	1.62	1.15	
Northern	*Cases*	6	4	10	5	17	7	+10.3% (-0.5% to +22.3%) [p=0.060]
	*ASR*	1.63	2.45	1.27	1.73	2.98	1.00	
	*CIR*	1.43	0.47	1.63	1.17	2.83	1.18	

## 3 Discussion

This study analyzed the trends in the incidence, stage distribution and regional distribution of HL in Saudi Arabia from 2011 to 2020, based on national registry data (20–29). This study is important because HL is one of the most prevalent cancers in Saudi Arabia (32). The 70.4% increase in HL cases over the decade can be attributed to a combination of the establishment of the SCR and a resultant improvement in diagnostic services and the reporting of such data to the registry.

Between 2011 and 2020, HL cases increased from 3.1% to 4.1% of all reported cancers, and the rising ASR (from 1.8 to 2.7) is consistent with the epidemiological transition seen in several middle- and high-income countries (4, 33). This increase could be a result of improved diagnostic precision, access to healthcare and enhanced completeness of cancer registries rather than representing a true rise in disease occurrence. The sharp increase between 2015 and 2016 coincides with national initiatives aimed at standardizing oncology reporting and strengthening the Saudi Cancer Registry (9). Globally, HL incidence has increased in developed countries, primarily among young adults, due to better health system access and awareness, whereas developing countries have shown a plateau (4). These findings highlight the importance of sustaining accurate and comprehensive cancer surveillance systems in order to determine true incidence rates over time.

The age-specific incidence in this study displayed a classical bimodal pattern, with peaks in adolescence and middle age, consistent with global data. The peak incidence in females occurred approximately five years later than in males. The initial peak (15–19 years in males; 20–24 years in females) reflects genetic susceptibility and delayed EBV infection, while the second peak in middle age may be attributed to immune, metabolic, or environmental risk factors (33). The male predominance observed aligns with international findings. The increase in HL among infants (0–5 years) in 2020 could be a result of improved early diagnosis rather than a true rise in incidence; however, this observation needs to be investigated further.

When morphological subtypes were studied, the analyses showed a predominance of HL NOS in both sexes, especially among males. The increase in HL NOS may reflect changes in pathological classification practices, pathology reporting protocols, incomplete subclassification, or potential true epidemiological transitions.

The reduction in HL mixed cellularity and nodular-lymphocyte predominance cases indicates epidemiological transitions linked to early detection, improved sanitation and reduced early-life EBV infection. Among females, declines in lymphocyte-rich and lymphocyte-depleted subtypes indicate a shift toward less aggressive morphologies, associated with improved population health and earlier detection (16).

Geographical variation was evident across Saudi Arabian regions. Najran and Tabuk exhibited the highest ASRs among males, increasing from 0.8 in 2011 to 7.4 per 100,000 in 2020 (Najran; +18.4% APC) and from 1.5 in 2011 to 6.5 in 2020 (Tabuk; +0.5% APC). In females, the highest increase was seen in Najran, Hail and the Eastern Province, whereas some provinces, such as Asir and Baha, showed a decrease in ASRs. Jazan’s consistently low ASR may reflect true epidemiological variation, reporting differences, or potential under-registration rather than a true low burden. These disparities likely reflect regional differences in diagnostic capacity, healthcare access, population density and environmental exposures. These results highlight the need for further investigation into these trends, which may consequently affect cancer control policies and resource allocation for early diagnosis and timely treatment.

In stage stratification, the increase in localized diagnoses (reaching 37.5%) serves as a promising metric of the impact of the Saudi Vision 2030 health transformation, which emphasizes the significance of early detection. There was a predominance of distant stage HL, which indicates that patients continue to present at advanced stages, a finding consistent with regional and international data (33, 34). Late presentation may arise from limited public awareness, delayed healthcare-seeking behavior, or diagnostic delays in peripheral regions. Early-stage diagnosis remains critical, as HL is among the most curable malignancies. The absence of stage data for 2019–2020 may be attributable to disruptions in the registry data collection during the COVID-19 pandemic, which also impacted cancer reporting globally.

This study identified a consistent upward trend in the incidence of HL and distinct regional, morphological and age-specific patterns. These trends need to be investigated further to guide necessary improvements in surveillance and addressing the changing patterns of the disease. Continued investment in diagnostic capacity, registry quality and early detection programs will be the key to reducing HL burden and improving outcomes. Saudi Arabia’s HL data reflect a transitional epidemiological profile, with geographical variations underscoring the need for sustained research and policy action.

Globally, HL accounted for approximately 0.4% of all new cancer cases and 0.2% of cancer deaths in 2020 (4). The rising incidence in Saudi Arabia parallels observations in other Gulf countries such as Kuwait, Bahrain and the UAE, where improvements in registry completeness and diagnostic accuracy have produced similar patterns (34). HL in Saudi Arabia presents at a slightly younger age than in the more developed regions, possibly as a result of genetic or infectious exposures. While incidence in the more developed countries has largely stabilized (4), the increase in incidence rates in a developing region such as Saudi Arabia underscores the need for a continued cancer surveillance.

### Study limitations

This study has several limitations. First, the reliance on aggregated secondary registry data restricted the capacity to conduct individual-level analyses or adjust for potential confounding variables. Second, the absence of stage data for the years 2019–2020 may have limited the interpretation of temporal stage trends. Finally, discrepancies in regional reporting completeness and diagnostic methodologies may have impacted the observed incidence patterns.

## 5 Conclusion

This study provides a decadal analysis of HL epidemiology in Saudi Arabia. The findings identified a consistent upward trend in incidence, along with distinct regional, morphological and age-specific patterns. These changes highlight the need for continued improvements in surveillance and strategies aimed at addressing the changing patterns of the disease. Continued investment in diagnostic capacity, registry quality and early detection programs will be key to reducing the HL burden and improving outcomes. Saudi Arabia’s data on HL reflect a transitional epidemiological profile, underscoring the need for sustained research and policy action.

## Data Availability

The datasets presented in this study can be found in online repositories. The names of the repository/repositories and accession number(s) can be found in the article/supplementary material.
